# Comparative Analysis of *Wolbachia* Genomes Reveals Streamlining and Divergence of Minimalist Two-Component Systems

**DOI:** 10.1534/g3.115.017137

**Published:** 2015-03-24

**Authors:** Steen Christensen, Laura Renee Serbus

**Affiliations:** Department of Biological Sciences, Florida International University, Miami, Florida 33199; Biomolecular Sciences Institute, Florida International University, Miami, Florida 33199

**Keywords:** *Wolbachia*, endosymbiont, α-proteobacteria, two-component signaling, genome organization

## Abstract

Two-component regulatory systems are commonly used by bacteria to coordinate intracellular responses with environmental cues. These systems are composed of functional protein pairs consisting of a sensor histidine kinase and cognate response regulator. In contrast to the well-studied *Caulobacter crescentus* system, which carries dozens of these pairs, the streamlined bacterial endosymbiont *Wolbachia pipientis* encodes only two pairs: CckA/CtrA and PleC/PleD. Here, we used bioinformatic tools to compare characterized two-component system relays from *C. crescentus*, the related Anaplasmataceae species *Anaplasma phagocytophilum* and *Ehrlichia chaffeensis*, and 12 sequenced *Wolbachia* strains. We found the core protein pairs and a subset of interacting partners to be highly conserved within Wolbachia and these other Anaplasmataceae. Genes involved in two-component signaling were positioned differently within the various *Wolbachia* genomes, whereas the local context of each gene was conserved. Unlike *Anaplasma* and *Ehrlichia*, *Wolbachia* two-component genes were more consistently found clustered with metabolic genes. The domain architecture and key functional residues standard for two-component system proteins were well-conserved in *Wolbachia*, although residues that specify cognate pairing diverged substantially from other Anaplasmataceae. These findings indicate that *Wolbachia* two-component signaling pairs share considerable functional overlap with other α-proteobacterial systems, whereas their divergence suggests the potential for regulatory differences and cross-talk.

Signaling mechanisms endow cells with the ability to sense and respond to environmental changes. One of the most-well studied types of signaling is that of two-component regulatory systems (TCSs), consisting of a sensor histidine kinase (HK) and paired response regulator (RR) (Mitrophanov and Groisman 2008; Capra and Laub 2012; Jung *et al.* 2012). TCS relays are the predominant form of signaling used in a majority of prokaryotes and can be found in fungi, slime molds, and plants as well (Krell *et al.* 2010; Stock *et al.* 2000; Grefen and Harter 2004; Capra and Laub 2012). A large body of research has determined that these sensor HKs are capable of recognizing stimuli such as oxygen, light, salinity, osmolarity, nutrients, or quorum sensing cues (Mascher *et al.* 2006). This leads to activation of cognate RRs, which coordinate a wide range of responses, including altering chemotaxis, activating sporulation, regulating bacterial differentiation, promoting binary fission, and regulating biofilm formation (Stock *et al.* 2000). TCSs have been found to regulate expression of genes that underlie key agricultural symbioses with *Rhizobium* and *Agrobacterium*, as well as virulence properties of pathogens like *Vibrio sp*., *Brucella sp*., and *Pseudomonas sp*. (Waters and Bassler 2005; Miller and Bassler 2001). In addition to positioning HK-RR pairs as desirable drug targets, this highlights the fundamental importance of TCS mechanisms.

The range of TCS proteins carried by each bacterium appears to correspond to the complexity of the bacterial life cycle. Some α-proteobacteria carry upwards of 100 HK and RR homologs, and the model system *Caulobacter crescentus*, which has a complex, dimorphic life cycle, encodes 62 HKs and 44 RRs (Galperin 2005; Purcell *et al.* 2008). In stark contrast, the obligate intracellular bacteria *Anaplasma phagocytophilum* and *Ehrlichia chaffeensis* have retained only 3 HKs and 3 RRs (Rikihisa 2010; Wakeel *et al.* 2010; Cheng *et al.* 2006; Kumagai *et al.* 2006; Lai *et al.* 2009). These are the TCS pairs CckA/CtrA, which coordinate gene expression and DNA replication, PleC/PleD, which drive synthesis of cyclic-di-guanosine monophosphate (*c-di*-GMP), and NtrY/NtrX, which coordinate nitrogen sensing with changes in gene expression (Laub *et al.* 2002; Skerker and Laub 2004; Jacobs-Wagner 2004; Paul *et al.* 2004; Aldridge *et al.* 2003; Pawlowski *et al.* 1991; Carrica *et al.* 2012). Studies have shown that HK/RR relationships are generally maintained through specific HK and RR residues that interface with one another (Skerker *et al.* 2008; Capra *et al.* 2012b). As such, insulation against cross-talk between HK/RR pairs is regarded as essential for maintaining function *in vivo* (Siryaporn and Goulian 2008; Groban *et al.* 2009; Laub and Goulian 2007). The conservation of these three specific TCS pairs highlights their importance as core environmental response mechanisms within the Anaplasmataceae family.

The mechanisms used by the core TCS proteins of Anaplasmataceae have been investigated in several bacterial systems. Cell-cycle kinase A (CckA) is referred to as a “hybrid” histidine kinase (Laub and Goulian 2007). It has an N-terminal sensor region neighbored by a central dimerization and phosphotransfer domain (DHp), an internal catalytic domain (CA), and a C-terminal REC domain (Supporting Information, Figure S1A). On activation, the CA domain of CckA transfers a phosphate from hydrolyzed ATP to a conserved histidine (His) in the DHp domain (Jacobs *et al.* 1999). This phosphate is ultimately transferred to an N-terminal REC domain in its cognate RR, in this case cell-cycle transcriptional regulator A (CtrA) (Jacobs *et al.* 1999). This phosphotransfer to the CtrA REC is facilitated by intermediary REC domains, including a C-terminal REC domain on CckA, and in some cases single REC domain proteins such as ChpT in *C. crescentus* (Biondi *et al.* 2006; Laub *et al.* 2007). Receipt of a phosphate by CtrA activates the function of its output domain, a helix-turn-helix (HTH) DNA-binding domain (Figure S1A). This enables CtrA to function in both transcriptional regulation and inhibition of chromosome replication (Laub *et al.* 2002; Skerker and Laub 2004).

By contrast, PleC and NtrY HKs are classified as “canonical” histidine kinases (Laub and Goulian 2007). These proteins carry an N-terminal sensor region, an internal DHp domain, and a C-terminal CA domain (Figure S1B). The CA phosphorylates the conserved His within the DHp, which transfers the phosphate to the cognate RR, PleD or NtrX, respectively (Lai *et al.* 2009; Kumagai *et al.* 2006). These RRs carry one or more REC domains with conserved aspartate (Asp) residues. Functional data suggest that the N-terminal REC has the most significant regulatory impact on the C-terminal output region of the RR (Lai *et al.* 2009; Gao *et al.* 2007). For PleD, that output region is a C-terminal GGDEF domain that synthesizes the important second messenger, *c-di*-GMP (Ryjenkov *et al.* 2005; Römling and Amikam 2006). For NtrX, that output domain has DNA-binding capacity, which enables it to act as a transcription factor for genes involved in nitrogen metabolism (Pawlowski *et al.* 1991; Cheng *et al.* 2014).

One of the most widespread Anaplasmataceae species is *Wolbachia pipientis*, present in 40% of all insect species as well as some filarial nematodes (Zug and Hammerstein 2012; Hedges *et al.* 2008; Cordaux *et al.* 2001; Taylor *et al.* 2005). Recent work has shown these bacterial endosymbionts to be closely linked with human health interests. *Wolbachia* underlie the neglected diseases African river blindness and lymphatic filariasis, which together threaten up to one-sixth of the world population (Hoerauf 2008; Saint Andre *et al.* 2002; Taylor *et al.* 2000). *Wolbachia* also suppress replication and transmission of RNA viruses in insects, including Dengue fever and Chikungunya (Teixeira *et al.* 2008; Hedges *et al.* 2008; Moreira *et al.* 2009). This raises a number of fundamental questions about *Wolbachia*–host interactions. How do *Wolbachia* respond to environmental cues? To what extent are TCS-related genes shared between *Wolbachia* genomes? Is there any evidence that putative TCS homologs are functional, and does variation between TCS genes in different *Wolbachia* strains help elucidate that function? TCS genes have previously been reported in *Wolbachia*, but very little is known about their function to date (Cheng *et al.* 2006; Brilli *et al.* 2010). Here, we investigate these questions, informed by publicly available bioinformatic data.

## Materials and Methods

### Identification of TCS-related homologs

All sequenced *Wolbachia* strains available in Genbank were initially assessed for completion (http://www.ncbi.nlm.nih.gov/genome/?term=wolbachia). Genomes documented as fully complete or near-complete were selected for further analysis and classified according to supergroup identity, as indicated by prior phylogenetic analyses (Table 1) (Cordaux *et al.* 2008). These genomes were individually searched for homology to deduced-TCS sequences using the NCBI-blastp server tool along with published information for *C. crescentus* HK and RR protein sequences (protein–protein BLAST; http://blast.ncbi.nlm.nih.gov/Blast.cgi) (Altschul *et al.* 1997, 2005). All such queries returned only CckA, PleC, CtrA, and PleD homologs. Full sequences of all *Wolbachia* TCS proteins were compared against *E. chaffeensis* homologs, and the resulting similarity/identity were compiled for the full sequences of all *Wolbachia* TCS proteins based on annotated *ab initio*:Prodigal 2.00 or GeneMarkS+ predictions. Components with known functional interaction to the TCS regulatory network in *C. crescentus* were also identified and homology searches were performed in a similar manner, identifying *Wolbachia* homologs for DivL, DnaA, CcrM, and ClpX/P. No other TCS-related homologs were identified, as per a cutoff e-value ≥1. Identity/similarity values to *E. chaffeensis* homologs were determined for all TCS-related proteins except CcrM, which was not found in other Anaplasmataceae species. Our results are consistent with other published data regarding the absence of NtrY/NtrX and single REC-phosphotransfer proteins (Brilli *et al.* 2010; Cheng *et al.* 2006).

**Table 1:** *Wolbachia* strains and supergroups analyzed in this study

*Wolbachia* strain	Host Type	Host	Supergroup	Genome Sequence Status*^a^*	Reference Sequence/Contig
*w*Oo	Worm	*Onchocerca ochengi*	C	Complete: annotated	HE660029.1
*w*Bm	Worm	*Brugia malayi*	D	Complete: annotated	AE017321.1
*w*Uni	Wasp	*Muscidifurax uniraptor*	A	Near complete/annotated	ACFP01000001-ACFP01000256
*w*Di	Psyllid	*Diaphorina citri*	B	Near complete/annotated	AMZJ01000001-AMZ01000124
*w*Pip Pel	Mosquito	*Culex quinquefasciatus Pel*	B	Complete: annotated	AM999887.1
*w*Pip JHB	Mosquito	*Culex quinquefasciatus JHB*	B	Near complete/annotated	ABZA01000001-ABZA01000021
*w*AlbB	Mosquito	*Aedes albopictus*	B	Near complete/annotated	CAGB01000001-CAGB01000165
*w*No	Fruit fly	*Drosophila simulans*	B	Complete: annotated	CP003883.1
*w*Ha	Fruit fly	*Drosophila simulans*	A	Complete: annotated	CP003884.1
*w*Ri	Fruit fly	*Drosophila simulans*	A	Complete: annotated	CP001391.1
*w*MelPop	Fruit fly	*Drosophila melanogaster*	A	Near complete/annotated	AQQE01000001-AQQE01000080
*w*Mel	Fruit fly	*Drosophila melanogaster*	A	Complete: annotated	AE017196.1

a As of November 2014.

### Genome alignments and operon predictions

**Figure 1:**
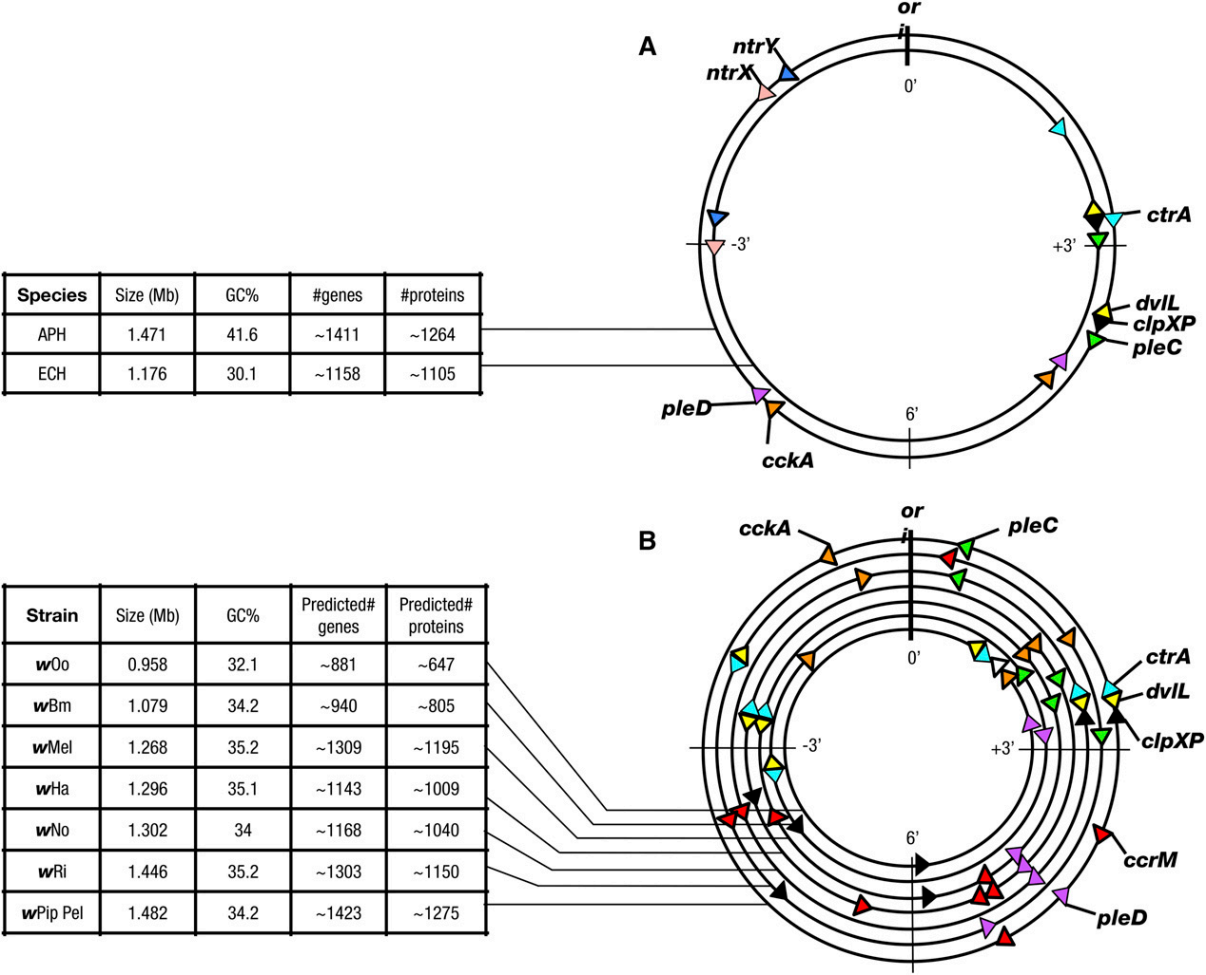
Syntenic alignments of the genomes from (A) *Anaplasma phagocytophilum* (*APH*) and *Ehrlichia chaffeensis* (*ECH*) and (B) various strains of *Wolbachia*. Representations of circular genomes are arranged in increasing size (not to scale). Arrows indicate relative genomic position, in minutes (±’; as o’clock position), and orientation of predicted ORFs in relation to *ori* (0′; arrowhead size also not to scale). Similarly colored triangles represent homologous ORFs, white triangle is the predicted *pleC* pseudogene for *w*Oo. Data in the associated tables, from NCBI genome reference information, are provided for comparison purposes.

Genomic positions for TCS genes and the associated ORFs of interest were determined for the completely sequenced and assembled *Wolbachia* strains *w*Oo, *w*Bm, *w*Mel, *w*Pip Pel, *w*Ha, *w*No, and *w*Ri, as well as for *A. phagocytophilum* and *E. chaffeensis*. First, the position and orientation of the origin of replication (*ori*) relative to *hemE* were identified (Ioannidis *et al.* 2007). Then, the distance between the first nucleotide position of each open reading frame (ORF) and the *ori* was calculated and set as a percentage of the total nucleotide size of each genome. The orientation of each ORF was also determined and positioned onto circular syntenic representations of each genome. Additional descriptive information for these genomes provided by Genbank (size, GC content, and estimates of gene/protein number) was included in Figure 1 for reference purposes.

Regions surrounding or adjacent to the identified TCS genes were further aligned using the Archaeal and Bacterial Synteny Explorer and using the “best genomic match” search parameter at a 10% minimal score threshold (http://archaea.u-psud.fr/absynte/) (Despalins *et al.* 2011). Scaled reproductions of these alignments were produced using information from the Arkin lab prokaryotic operon predictions program (www.microbesonline.org) and the program DOOR: Database of prOkaryotic OpeRons (http://csbl.bmb.uga.edu/DOOR/) (Price *et al.* 2005a,b; Dam *et al.* 2007; Mao *et al.* 2009). Statistical calls regarding the probability of operon structure were used to guide color-coding of ORFs. Cross-referencing of overlapping data sets from both programs was used to confirm predictions when available.

For CtrA binding site identification, perfect matches to the consensus α-proteobacterial CtrA binding site 8-mer (TTAACCAT) and 9-mer (TTAA-N7-TTAAC) sequences were identified on + or − strands, using the “find” function in CLC Sequence Viewer (version 7.5) (Brilli *et al.* 2010; Cheng *et al.* 2011). Fully sequenced *Wolbachia* genomes were used as input and site matches within −450 base pairs of the start of translation, defined by annotated ORF predictions, were selected as hits. Hits outside of these upstream regions were noted and are included in the total number of sites. Consensus sites contained within a previous ORF or positioned exactly at the starting nucleotide are included in the total number of sites for each strain. CcrM methylation sites were identified according to the consensus GANTC (Brilli *et al.* 2010; Stephens *et al.* 1996).

### Locus sequence confirmation

GenBank-deposited ORF predictions specifically for *pleD* from *w*Oo, and *cckA* of *w*AlbB and *w*Mel strains, were confirmed using the alignment function of CLC sequence viewer (version 6.9.1; http://CLCbio.com). To confirm the *w*AlbB cckA sequence, genomic DNA samples of *w*AlbB were collected from Sau5B mosquito tissue culture cells and *w*AlbB-infected *A. albopictus* mosquitoes, kindly provided by Jason Rasgon, Pennsylvania State University. The DNeasy Blood and Tissue extraction kit was used to extract purified DNA (Qiagen, Louisville, KY). *Wolbachia* DNA was also harvested from several *Wolbachia*-infected *Drosophila* stocks using the same method. *D. melanogaster* stocks of the genotype *w*; *Sp/Cyo*; *Sb/TM6B* were used, which had been infected previously with the *w*MelPop or *w*Mel *Wolbachia* strains (Serbus and Sullivan 2007). Independent lines of *D. simulans* carrying either *w*Ri or *w*Mel *Wolbachia* were also used (Veneti *et al.* 2003).

Full-length *cckA* was then PCR-amplified from fly-host *Wolbachia* samples with forward 5′-AAGGAACTTAATTAGATTTGGATG and reverse 5′-AGCAAAGGCTGTCGAYAAAT primers using FlexiTaq DNA polymerase according to manufacturer’s protocol (Promega, Madison, WI). For *w*AlbB, *cckA* fragments were PCR-amplified from both tissue culture and whole mosquito DNA samples using forward 5′-AAGGAAGCGATTGAACATGG and reverse 5′-AGCAAAGGCTGTYGAYAAAT primers. Thirty rounds of PCR were performed at an annealing temperature of 56° for 30 sec and product extension was performed at 72° for 2 min. Resulting PCR fragments were analyzed on a 1% agarose gel and prepared for sequencing using ExoSAPIT according to the manufacturer’s protocol (Affymetrix, Santa Clara, CA). ABI BigDye (R) Terminator v3.1 cycle sequencing reactions using the terminal forward and reverse primers, as well as specific internal primers, were analyzed on an ABI 3100 Genetic Analyzer with sequencing analysis and Genescan software (Applied Biosystems, CA). Coverage of greater than 6× was obtained for each sequence, and nucleotide identities were manually checked against alignments. Sequence information for the entire *pleD* region from each of the *Wolbachia* fly–host combinations was also obtained, confirming deposited sequences.

### Alignments, domain architecture, and cognate residue identification

The deduced amino acid sequences of predicted TCS ORFs in *C. crescentus*, *A. phagocytophilum*, *E. chaffeensis*, and all *Wolbachia* strains were complied and cross-referenced to CLC Sequence Viewer-deduced sequences (version 6.9.1; http://CLCbio.com). Corresponding protein accessions, annotated lengths, and percent identity/similarity to *E. chaffeensis* homologs were compiled. Domain structure and conserved motifs/residues were then identified using Pfam database annotations and the Simple Modular Architecture Research Tool (SMART; http://smart.embl-heidelberg.de/) (Schultz *et al.* 1998; Letunic *et al.* 2012). These tools returned similarly significant e-values for the signaling-associated DHp, CA, and REC domains (Conserved Domain Database entries CDD119399, CDD238030, and CDD238088, respectively). Phospho-transfer and phospho-acceptor sites, as well as residues needed to confirm kinase/phosphatase-specific function, were identified by homology to Pfam annotations. Catalytic domain-specific Mg^2+^ binding sites were identified similarly. The HTH domain and the DNA recognition α3 helix were both identified by comparison against the conserved α-proteobacterial CtrA orthologs (Martinez-Hackert and Stock 1997; Quon *et al.* 1996; Lang and Beatty 2000; Bird and Mackrell 2011). For PleD homologs, all residues that form the active site, the metal-binding site, and I-site of the GGDEF domain were marked according to the Conserved Domains Database annotations for *E. chaffeensis* (CDD:143653) (Chan *et al.* 2004; Christen *et al.* 2006).

Deduced amino acid alignments were generated using the “create alignment” function of CLC-sequence viewer 6.9.1 based on the CLUSTALW alignment matrix/algorithm. The domains, residues, and sites described above were manually marked on the alignments. The positions of HK/RR cognate specificity residues were identified by comparison against *C. crescentus*. Additional alignments using the *E. coli* EnvZ histidine kinase and *B. subtillus* OmpR response regulator were also used to verify the alignment and cognate residue positioning for each TCS component (Skerker *et al.* 2008; Capra *et al.* 2012a). Comparisons between cognate-specifying residues on the DHp and its corresponding REC were then evaluated for covariation against their *E. chaffeensis* homologs.

The predictions of the transmembrane regions and PAS-associated domains for the N-terminal halves of CckA and PleC varied substantially between *Wolbachia* strains according to SMART/BLAST alignment analysis. Thus, we used the TransMembrane Helix Markov Model website (TMHMM Server 2.0; http://www.cbs.dtu.dk/services/TMHMM/) to determine the probability of membrane spanning helixes (to a cut-off of *P* = 0.8) as well as the Phyre 2.0 server (http://www.sbg.bio.ic.ac.uk/phyre2) to determine the likelihood of secondary structure formation consistent with other predictions (Krogh *et al.* 2001; Kelley and Sternberg 2009). Because Phyre 2.0 predictions for PAS-like folds in *C. crescentus* CckA and DivL sequences were consistent with both BLAST-identified PAS domain e-value predictions and published results, this indicated Phyre to be a valid tool for predicting the presence of PAS-like folds.

First, the N-terminal halves of CckA sequences were submitted, followed by defined regions potentially containing PAS domains. This revealed the classic 5-beta strand PAS-fold feature for all PAS-like domains in *Wolbachia* DvlL and CckA homologs, with notable variation in supergroups A and B *Wolbachia* CckA homologs. The PSIPRED Protein Sequence Analysis Workbench (http://bioinf.cs.ucl.ac.uk/psipred/) was used to further investigate PAS-domain secondary structure predictions in *Wolbachia* CckA. This program confirmed alpha-helix and beta-strand predictions consistent with the classic 5-beta strand PAS-fold for CckA from all *Wolbachia* strains. Additional ligand-binding potential was indicated by the Phyre 2.0 3DLigandSite server (http://www.sbg.bio.ic.ac.uk/3dligandsite/) for which confidence values had an average *Ln*E ≥10 for all PAS domains (average *Ln*E range of 9.0-13.4, with a value of >4.0 considered significant) (Wass *et al.* 2010). The resulting domain architecture was graphically represented.

## Results

### Identification of core TCS genes in *Wolbachia pipientis*

The widespread use of TCS by eubacteria raises the question of how widely these genes have been retained in endosymbiotic *Wolbachia* bacteria. Prior studies indicate that the *Wolbachia* relatives *A. phagocytophilum* and *E. chaffeensis* carry the TCS pairs: *cckA*/*ctrA*, *pleC*/*pleD* and *ntrY*/*ntrX* (Kumagai *et al.* 2006; Lai *et al.* 2009). This annotation is based on deduced amino acid sequences, which exhibit 55–67% similarity to the TCS homologs in *C. crescentus* (Table S1). In accordance with this, we used predicted amino acid sequences from the closer phylogenetic relative, *E. chaffeensis*, to identify TCS homologs in *Wolbachia* (Brouqui and Matsumoto 2007). We searched the genomes of 12 completely or near completely sequenced *Wolbachia* strains, which are classified in supergroups A–D, and represent symbiosis with a range of insect and nematode hosts (Table 1). This revealed that, in addition to a few previously examined strains, all *Wolbachia* lack detectable homologs for *ntrY* and *ntrX*, whereas corresponding homologs for the four other TCS genes were ubiquitously detected (Table 2). One of the exceptions was *w*Oo, in which *pleC* is annotated as a pseudogene. In three other cases, a single TCS gene is predicted to be split into multiple open reading frames (ORFs). This is seen for *pleD* of *w*Oo as well as for *cckA* of *w*AlbB and *w*Mel.

**Table 2:** TCS gene name, protein accession number, length, e-value, and amino acid identity/similarity to *Ehrlichia* homolog

*Wolbachia* Strain	*cckA*	*ctrA*	*pleC*	*pleD*	*dvlL*
*ECH*	YP_507553.1 (828 aa)	YP_507798.1 (256 aa)	YP_507680.1 (470 aa)	YP_507571.1 (458 aa)	YP_507699.1 (381 aa)
	(DHp-CA region)*^a^*	(entire sequence)*^a^*	(DHp-CA region)*^a^*	(entire sequence)*^a^*	(entire sequence)*^a^*
*w*Oo	wOo_05930	wOo_05460	[wOo_05520]	wOo_06950-60*^b^*	wOo_05420
	CCF78223 (826 aa)	CCF78193 (250 aa)	[pseudogene]	CCF78286-71 (311 aa)	CCF78189 (378 aa)
	0.0 (67%/80%)	1e-120 (71%/85%)	[pseudogene]	6e-101 (57%/77%)	5e-101 (43%/65%)
*w*Bm	Wbm0710	Wbm0596	Wbm0128	Wbm0184	Wbm0599
	AAW71298 (826 aa)	AAW71184 (256 aa)	AAW70719 (475 aa)	AAW70775 (458 aa)	AAW71187 (378 aa)
	0.0 (67%/80%)	6e-122 (72%/85%)	6e-142 (61%/82%)	8e-164 (58%/78%)	1e-102 (44%/66%)
*w*Uni	WUni_006980	WUni_002760	WUni_005930	WUni_003350	—
	EEH11963 (826 aa)	EEH12358 (256 aa)	EEH12088 (472 aa)	EEH12305 (460 aa)	—
	0.0 (68%/80%)	1e-122 (71%/85%)	5e-138 (61%/82%)	0.0 (57%/76%)	—
*w*Di	WDIAC_01745	WDIAC_03145	WDIAC_03885	WDIAC_00280	WDIAC_03125
	WP_017531904 (826 aa)	WP_017532132 (256 aa)	WP_017532256 (475 aa)	WP_017531661 (457 aa)	WP_017532129 (378 aa)
	0.0 (67%/80%)	1e-122 (70%/84%)	5e-138 (61%/82%)	5e-138 (58%/78%)	2e-100 (43%/63%)
*w*Pip Pel	WPa_0966	WPa_0585	WPa_0784	WPa_0358	WPa_0581
	YP_001975718 (826 aa)	YP_001975355 (256 aa)	YP_001975544 (475 aa)	YP_001975155 (458 aa)	YP_001975351 (378 aa)
	0.0 (67%/80%)	2e-116 (69%/83%)	2e-129 (57%/80%)	0.0 (58%/78%)	5e-100 (43%/63%)
*w*Pip JHB	C1A_531	C1A_168	C1A_361	C1A_1169	C1A_164
	EEB56349 (826 aa)	EEB55578 (256 aa)	EEB56179 (444 aa)	EEB55360 (458 aa)	EEB55574 (378 aa)
	0.0 (67%/80%)	2e-116 (69%/83%)	1e-129 (57%/80%)	0.0 (58%/78%)	5e-100 (43%/63%)
*w*AlbB	WALBB_620009-10*^b^*	WALBB_700001	WALBB_150003	WALBB_100006	WALBB_690007
	CCE77611 (744 aa)	CCE77711 (256 aa)	CCE77185 (468 aa)	CCE76884 (458 aa)	CCE77692 (378 aa)
	0.0 (67%/80%)	w9e-118 (70%/84%)	6e-130 (61%/82%)	8e-170 (58%/78%)	6e-101 (43%/63%)
*w*No	wNo_05610	wNo_02870	wNo_04460	wNo_00860	wNo_02830
	AGJ98979 (826 aa)	AGJ98722 (256 aa)	AGJ98870 (475 aa)	AGJ98539 (458 aa)	AGJ98718 (378 aa)
	0.0 (66%/80%)	4e-118 (70%/84%)	2e-126 (57%/80%)	6e-171 (58%/78%)	5e-100 (43%/63%)
*w*Ha	wHa_10160	wHa_06210	wHa10690	wHa_01880	wHa_06180
	AGK00427 (826 aa)	AGK00064 (256 aa)	AGK00478 (475 aa)	AGJ99670 (460 aa)	AGK00061 (378 aa)
	0.0 (68%/80%)	7e-122 (72%/85%)	1e-137 (61%/82%)	5e-170 (57%/76%)	9e-97 (41%/63%)
*w*Ri	WRi_011950	WRi_007440	WRi_013110	WRi_002100	WRi_007480
	ACN95881 (826 aa)	ACN95493 (256 aa)	ACN95987 (475 aa)	ACN95041 (458 aa)	ACN95497 (378 aa)
	0.0 (68%/80%)	7e-122 (72%/85%)	4e-138 (61%/82%)	0.0 (59%/76%)	3e-95 (41%/63%)
*w*MelPop	WMELPOP_00349	WMELPOP_03997	WMELPOP_00647	WMELPOP_01748	WMELPOP_03977
	ERN56258 (826 aa)	ERN55516 (256 aa)	ERN56200 (468 aa)	ERN55951 (460 aa)	ERN55512 (378 aa)
	0.0 (68%/80%)	5e-122 (72%/85%)	2e-140 (61%/82%)	0.0 (58%/76%)	9e-97 (41%/63%)
*w*Mel	WD_1215-16*	WD_0732	WD_1284	WD_0221	WD_0728
	NP_966927-8 (826 aa)*^c^*	NP_966490 (256 aa)	NP_966994 (475 aa)	NP_966031 (460 aa)	NP_966486 (378 aa)
	0.0 (68%/80%)	5e-122 (72%/85%)	2e-140 (61%/82%)	1e-170 (58%/76%)	9e-97 (41%/63%)

a e-values using Wolbacheae organism data-set cutoff in NCBI Bacterial genome BLAST; % identity/%similarity based on *ECH* sequence or region indicated.

^–^Genome sequence incomplete; nearest contig ends before the start of *dvlL* ORF.

b Multiple ORFs; e-value is for longest ORF (wOo_06950 and WALBB_620009)

c Accessions based on Genbank entries for this region; deduced amino acid length and comparison values based on nucleotide information in Figure S2.

A split ORF in any *Wolbachia* TCS gene could dramatically affect signaling processes in a system lacking functionally redundant genes. To confirm the basis for the split ORF predictions, we re-examined the deposited sequences of *w*Oo *pleD*, *w*AlbB *cckA*, and *w*Mel *cckA* genes. For *w*Oo *pleD*, nucleotide sequence alignments and visual inspection revealed multiple nucleotide substitutions leading to four stop codons between wOo_06950 and wOo_06960. A frameshift was also detected that positions these ORFs in different reading frames. Because these data cannot be substantiated by any single sequencing error in relation to other *Wolbachia pleD* genes, these findings are consistent with a split ORF prediction in the *w*Oo *pleD* locus.

Investigating the basis for the prediction in *w*AlbB *cckA* locus revealed five in-frame stop codons, partitioning the gene into two annotated ORFs, WALBB_620009 and WALBB_620010. Because all of these changes could be attributed to a single nucleotide deletion, it was unclear whether this change was genuine or reflected an artifact in the deposited sequence. Our re-sequencing of this *cckA* region, using *w*AlbB DNA isolated from both *A. albopictus* tissue culture cells and intact mosquitoes, revealed an exact match with the deposited sequence. Thus, data obtained from our two independent samples confirm the split ORF prediction for *w*AlbB *cckA*.

Analysis of the genomic region for *w*Mel *cckA* also indicated that the split ORF prediction was potentially attributable to a single nucleotide addition in the deposited sequence, creating a stop codon that partitioned *w*Mel CckA into the ORFs WD1215 and WD1216. To verify whether this split ORF prediction is accurate, we sequenced *cckA* of *w*Mel carried by *D. melanogaster* (Serbus and Sullivan 2007) and in a transinfected *D. simulans* strain (Poinsot *et al.* 1998). As controls, *cckA* was also sequenced from *w*MelPop and *w*Ri, attained from lab strains of *D. melanogaster* and *D. simulans*, respectively. We found that the ∼2.5-kB fragment sequenced from *w*MelPop and *w*Ri *cckA* exactly matched the Genbank record. This was also the case for nearly all of the *w*Mel *cckA* sequence from both *Drosophila* hosts, including the *w*Mel-associated SNP found at position 2402 (Chrostek *et al.* 2013). However, both of the re-sequenced *w*Mel *cckA* samples lacked the frame-shifting cytosine at position 1149 of the deposited *w*Mel *cckA* sequence (Figure S2). This indicates that *w*Mel *cckA* is more likely encoded by a single ORF, analogous to *cckA* in other *Wolbachia* strains. Further analysis of *w*Mel CckA, presented below, is done in accordance with this finding.

### Identification of TCS-related genes in *Wolbachia pipientis*

The presence of TCS genes in *Wolbachia* raises other questions about how well the overall TCS regulatory network is conserved. In the *Caulobacter* system, a complex network of kinases and phosphotransfer proteins affects the signaling ability of CckA and PleC (Ausmees and Jacobs-Wagner 2003; Biondi *et al.* 2006). These include DivL, an HK-related tyrosine kinase that promotes CckA signaling; ChpT, an intermediary phosphotransfer protein; CpdR and DivK, response regulators that can also interact with CckA; and DivJ, an HK whose activity directly opposes that of PleC. No homologs for *chpT*, *cpdR*, *divK*, or *divJ* have been reported for *Anaplasma* or *Ehrlichia*, and our analyses did not identify homologs in *Wolbachia* (Brilli *et al.* 2010). However, coding sequence homologous to *Caulobacter divL* was widely shared between the *Anaplasmataceae* and *Wolbachia* (Table 2, Table S1). This sequence, encoding an approximately 400-amino-acid-long N-terminal fragment of DivL, will be referred to as *dvlL* (for DivL-like) in this analysis. *A. phagocytophilum*, *E. chaffeensis*, and 11 of 12 *Wolbachia* strains analyzed all contained *dvlL*. The status of *dvlL* was inconclusive in the *w*Uni *Wolbachia* strain due to lack of sequence coverage in that region of the genome (Table 2). The importance of DivL in well-characterized bacterial systems and the conservation of *dvlL* in *Wolbachia* open the possibility that DvlL interacts with other *Wolbachia* TCS components.

α-Proteobacteria are known to carry other factors that modulate CtrA activity as well (Christen *et al.* 2006; McGrath *et al.* 2006; Gorbatyuk and Marczynski 2005). These include CcrM, a methyltransferase that modifies the *ctrA* promoter region; GcrA, a transcriptional activator of *ctrA*; and SciP, a transcriptional repressor of CtrA-regulated genes. Neither *Anaplasma* nor *Ehrlichia* has been reported to carry homologs for *ccrM*, *gcrA*, or *sciP* (Brilli *et al.* 2010; Tan *et al.* 2010; Fioravanti *et al.* 2013; Stephens *et al.* 1996). However, the majority of sequenced mosquito and fruit fly *Wolbachia* strains contained anywhere from one to three copies of the *ccrM* gene (Table S2). Because these strains also carried 2 CcrM methylation sites within 400 base pairs of the *ctrA* start site (unpublished observation), the presence of *ccrM* has possible implications for *Wolbachia* TCS and cell cycle regulation.

Many α-proteobacteria have been shown to use additional regulatory proteins to drive shutdown of CtrA and PleD outputs through degradation (Christen *et al.* 2006; McGrath *et al.* 2006; Gorbatyuk and Marczynski 2005). These include ClpX/P, a protease that degrades CtrA, clearing the origin of replication (*ori*) for DnaA to bind and initiate DNA replication; RcdA and PopA, which facilitate CtrA interaction with ClpX/P; and EAL-domain phosphodiesterase proteins, which hydrolyze the *c-di*-GMP second messenger produced by PleD (McGrath *et al.* 2006; Ryan *et al.* 2004; Jenal and Fuchs 1998; Simm *et al.* 2004; Christen *et al.* 2005). Consistent with prior analyses of other Anaplasmataceae, *rcdA*, *popA*, and any EAL domain–encoding genes could not be identified in sequenced *Wolbachia* strains (Taylor *et al.* 2009; Ozaki *et al.* 2014; Cheng *et al.* 2006). However, homologs were identified for *clpX* and *clpP*, as well as for *dnaA* in 12 of 12 sequenced *Wolbachia* strains (Table S1, Table S2). These results taken together indicate that *Wolbachia* have retained a subset of factors that regulate TCS activity.

### Genome-wide positioning of TCS-related genes in *Wolbachia pipientis*

The positioning of genes throughout the bacterial genome has a strong impact on relative expression throughout the cell cycle (Condon *et al.* 1992). Given the evidence that *Wolbachia* share core TCS-related genes with *Anaplasma* and *Ehrlichia*, we asked whether the overall positioning of these genes is also conserved in *Wolbachia*. To address this, we created syntenic alignments using the genomes of completely assembled *Wolbachia* strains. These were aligned with respect to the *ori* locus and oriented according to the proximal *hemE* gene (Ioannidis *et al.* 2007). The relative positions of conserved TCS-related genes were then plotted on this map, with the *ori* for all genomes shown at position 0′ and the terminus at the relative position of 6′ (Figure 1, Table S3).

This analysis indicated that a subset of TCS-related genes was similarly positioned with respect to the *ori* in *A. phagocytophilum*, *E. chaffeensis*, and *Wolbachia*. This includes *ctrA*, positioned approximately 2′–3′ distant from the *ori*, *dvlL*, closely associated with *ctrA* in *Wolbachia*; and *pleD*, positioned approximately 3′–5′ from the *ori*. Positioning trends for *cckA*, *pleC*, and *clpX/P* were also visible between *A. phagocytophilum* and *E. chaffeensis*, as well as between *Wolbachia* strains, but not between the three genera collectively. Copies of the *ccrM* gene, absent from *A. phagocytophilium* and *E. chaffeensis*, were generally positioned 4′–5′ distant from the *ori* in fly and mosquito *Wolbachia* strains. *Wolbachia cckA* and *pleC* were positioned closer to the *ori*, whereas *clpX/P* was positioned more distantly than in *A. phagocytophilium* and *E. chaffeensis*. In addition, the clustering of *dvlL* and *clpX/P* genes seen in *A. phagocytophilium* and *E. chaffeensis* was not shared by the *Wolbachia* genomes, which consistently showed *dvlL* proximal to the *ctrA* locus (Figure 1, Table S3). This differential positioning raises the possibility that *Wolbachia* TCS gene dosage may differ appreciably from *A. phagocytophilum* and *E. chaffeensis* during the cell cycle (Couturier and Rocha 2006).

### Immediate context of the core *Wolbachia* TCS genes

**Figure 2:**
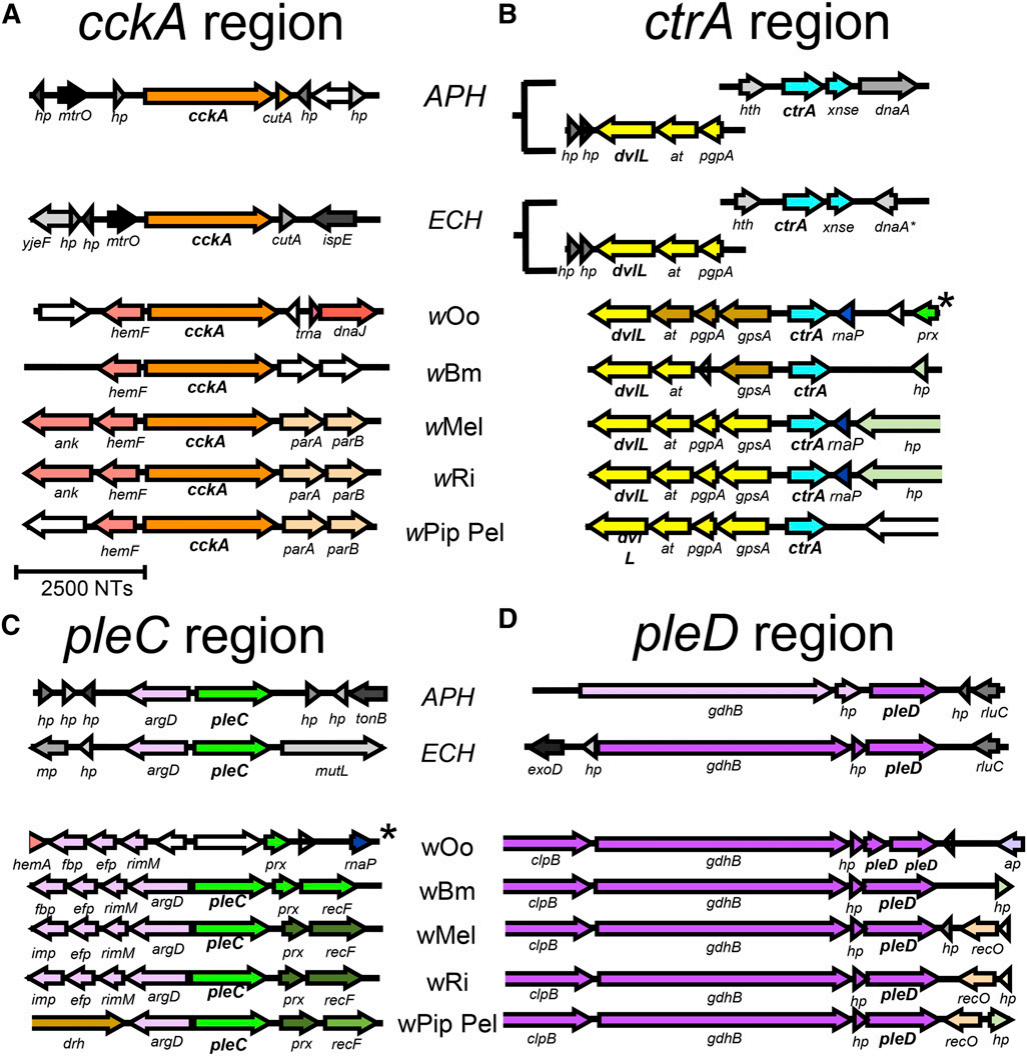
Synteny and operon predictions for the genetic regions surrounding the (A) *cckA*-, (B) *ctrA*-, (C) *pleC*-, and (D) *pleD*- genes for *A. phagocytophilum* (*APH*), *E. chaffeensis* (*ECH*), and the *Wolbachia* strains indicated. Each line represents a genetic region from the organism/strain indicated. *Region surrounding *ctrA* in *w*Oo is adjacent to the region surrounding the predicted pseudogene for pleC. Color-filled arrows are predicted ORFs in their respective orientations; white arrows are predicted pseudogenes. Similarly colored arrows are ORFs predicted to share a common operon based on data from Table S4; open arrows indicate an ORF that extends beyond the region shown. Gene names are referenced along with locus tag information in Table S4.

To further evaluate the genomic context immediately flanking the TCS genes of *A. phagocytophilum*, *E. chaffeensis*, and *Wolbachia*, we aligned these regions and analyzed them with several operon prediction programs (Table S4) (Price *et al.* 2005a,b; Dam *et al.* 2007; Mao *et al.* 2009). This revealed some variation in the context of all shared TCS loci. For *A. phagocytophilum* and *E. chaffeensis*, the *cckA* gene was closely flanked by the genes *o-methyltransferase* and *cutA*, which encode a cation tolerance protein (Figure 2A). However, *cckA* in all *Wolbachia* strains was neighbored at its 5′ end by the *hemF* gene, which supports heme biosynthesis (Heinemann *et al.* 2008). Furthermore, all sequenced *Wolbachia* genomes, except the phylogenetically distant strains *w*Bm and *w*Oo, showed *cckA* as being flanked at its 3′ end by *parA* and *parB*, which encode chromosomal partitioning proteins (Figure 2A) (Foster *et al.* 2005).

A similar type of contextual variation was evident for *Wolbachia ctrA*. In *A. phagocytophilum* and *E. chaffeensis*, *ctrA* was flanked upstream by a gene encoding a helix-turn-helix (*hth*) DNA binding protein and downstream by *xnse*, which encodes a 3′–5′ exonuclease family protein (Figure 2B). However, in nearly all sequenced *Wolbachia* strains, *ctrA* appeared to share an upstream region with an operon that contains *dvlL*, as well as the genes *glycerol-3-phosphate dehydrogenase*, *phosphotidylglycerophosphatase A*, and an *acetyltransferase* (Table S4). This genomic arrangement was similar in *wBm* and *wOo*, although the neighboring operon may be fragmented or incomplete. The 3′ end of *Wolbachia ctrA* was flanked by a variety of genetic regions that differed according to supergroup (Figure 2B). Thus, the genomic context of *cckA* and *ctrA* is generally conserved between *Wolbachia* strains, although not between *Wolbachia* and other Anaplasmataceae.

In contrast, the immediate context of *pleC* and *pleD* appeared relatively more conserved. Analysis of the *A. phagocytophilum* and *E. chaffeensis pleC* region suggested that *pleC* shares a promoter with the nitrogen metabolism gene *argD* (Velasco *et al.* 2002), with its 3′ end flanked by either hypothetical genes or the *mutL* membrane protein gene (Figure 2C). Interestingly, in all sequenced *Wolbachia* genomes except *w*Oo, which lacks detectable homologs for both genes, *pleC* ORFs were predicted to share a promoter with *argD*, analogous to *Anaplasma* and *Ehrlichia*. However, *Wolbachia pleC* was also flanked by *peroxiredoxin* and the recombination gene *recF* at its 3′ end, indicating that the *pleC* genomic region is not entirely conserved (Figure 2C).

Examination of the *pleD* region suggested a similar extent of conservation between species. In *A. phagocytophilum* and *E. chaffeensis*, *pleD* was neighbored at the 5′ end by *glutamate dehydrogenase B* and a short hypothetical protein ORF denoted as *hp* (Figure 2D). This *gdhB-hp-pleD* cluster was predicted to form an operon in *Ehrlichia* (Table S4). Interestingly, a *gdhB-hp-pleD*–containing operon was also consistently predicted in *Wolbachia*, with the addition of a chaperonin gene, *clpB*, included at the 5′ end of the operon (Figure 2D). Thus, considerable homology is evident in the genomic context of *pleC* and *pleD* among *Wolbachia* strains, some of which is shared with other Anaplasmataceae.

### Comparison of domain structure between TCS homologs

**Figure 3:**
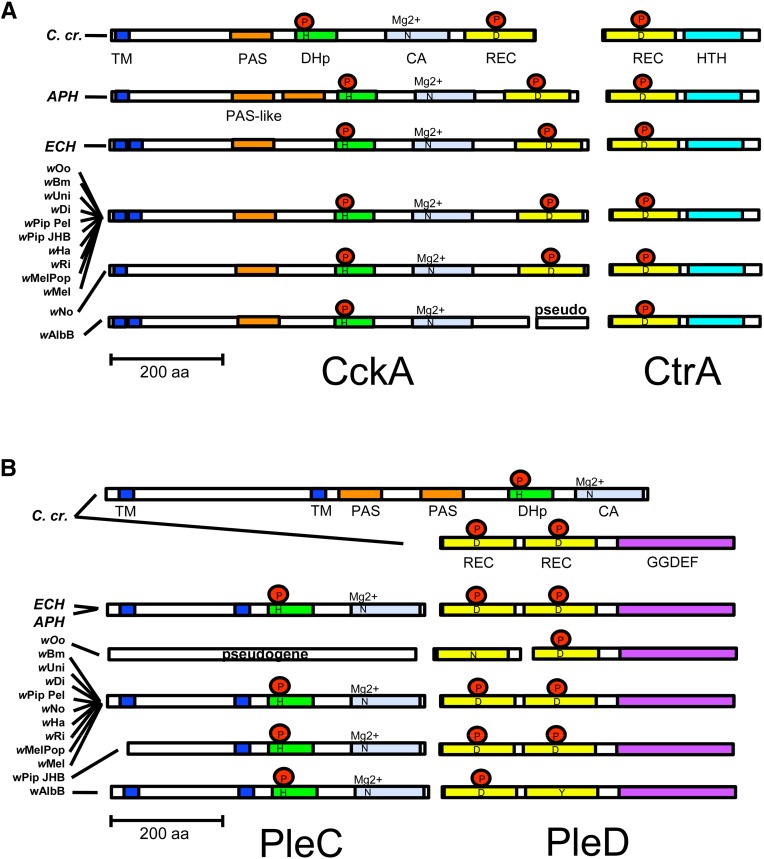
Domain architecture of deduced amino acid sequence for the TCS pairs (A) CckA/CtrA and (B) PleC/PleD. For comparison, *C. cresentus* (*C.cr*.) domains, as well as those from *A. phagocytophilum* (*APH*) and *E. chaffensis* (*ECH*), are shown. *w*Mel is represented by predicted architecture for the independently sequenced strains from this study. CA, catalytic-ATPase domain; CC, coiled-coil; DHp, dimerization and histidine-phosphotransfer domain; GGDEF, di-guanylate cyclase domain; HTH, helix-turn-helix DNA-binding domain; PAS, P(er) A(rnt) S(im)-like sensor domain fold; REC, response-receiver domain; TM, *trans*-membrane region; D, aspartate; H, histidine; Mg^2+^, magnesium; N, asparagine; P, phosphate; Y, tyrosine

**Figure 4:**
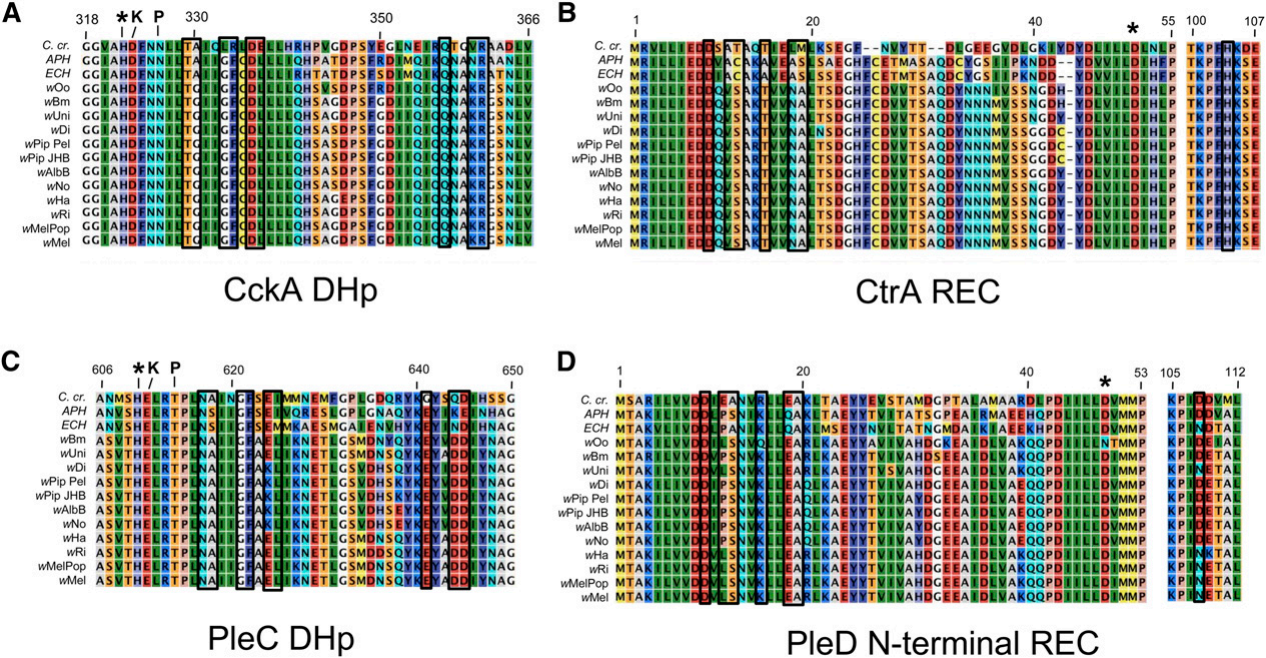
Analysis of CckA/CtrA and PleC/PleD cognate pairing-residue alignments. (A) CckA HK domain, (B) CtrA REC domain, (C) PleC DHp, and (D) the PleD N-terminal REC domain. Identical/similar residues are similarly colored. Amino acid numbers shown above are for the *C. cresentus* sequence. Asterisk indicates conserved phosphorylation sites, K indicates residue necessary for kinase function, P indicates residue necessary for phosphatase function (Willett and Kirby 2012). Boxed residues in alignments indicate covarying residues critical in specifying cognate HK/RR interaction (Capra *et al.*, 2012a; Podgornaia and Laub 2013).

If *Wolbachia* TCS proteins are functional, then the predicted products should carry the domains and key residues important for activity. To resolve this issue, we compared the predicted functional domains of the *Caulobacter* TCS proteins against *A. phagocytophilum*, *E. chaffeensis*, and *Wolbachia. A. phagocytophilum* and *E. chaffeensis* CckA exhibited features typical of a hybrid-HK (Figure 3A, Figure S1) (Dutta *et al.* 1999). The N-terminal sensor region of CckA contained a transmembrane domain, followed by a region of predicted secondary structure indicating classic PAS-fold architecture (see *Materials and Methods*; Table S5, Table S6). Two of these “PAS-like” domains were found in *A. phagocytophilum* and one was found in *E. chaffeensis*. Neighboring this N-terminal “sensor” portion, a dimerization/histidine phosphotransfer (DHp) domain was predicted. The DHp contained the conserved His residue, as well as two closely flanking residues that impart both kinase and phosphatase capabilities to the DHp (Figure 4A) (Willett and Kirby 2012). Following the DHp domain was an internal ATP-catalysis domain (CA) with a conserved asparagine (Asn), and a C-terminal REC domain with a conserved Asp (Figure 3A, Table S5) (West and Stock 2001).

Analogous to other Anaplasmataceae, most *Wolbachia* CckAs were predicted to carry internal DHp and CA domains, a C-terminal REC domain, and all the key functional residues associated with those domains (Figure 3A, Table S5) (Cheng *et al.* 2006; Kumagai *et al.* 2006). One exception to this was *w*AlbB, truncated partway into the C-terminal REC due to a split ORF and lacking the conserved Asp residue, confirmed by our sequencing results. All *Wolbachia* CckAs were predicted to have two N-terminal transmembrane domains, except *w*No. Predicted secondary structures also indicated that all *Wolbachia* CckAs carried at least one PAS-like domain (Figure 3A, Table S5, Table S6). The conservation of these structural features suggests a functional role for CckA has been conserved in *Wolbachia*. Furthermore, examination of DvlL domain structure indicated the three previously identified PAS domains, as well as complete conservation of DvlL between all *Wolbachia* strains (Figure S3, Table S5) (Childers *et al.* 2014). This raises the possibility that CckA regulation, as seen in the well-defined free-living α-proteobacterial model *Caulobacter*, may be at least partly conserved in *Wolbachia* as well.

**Figure 5:**
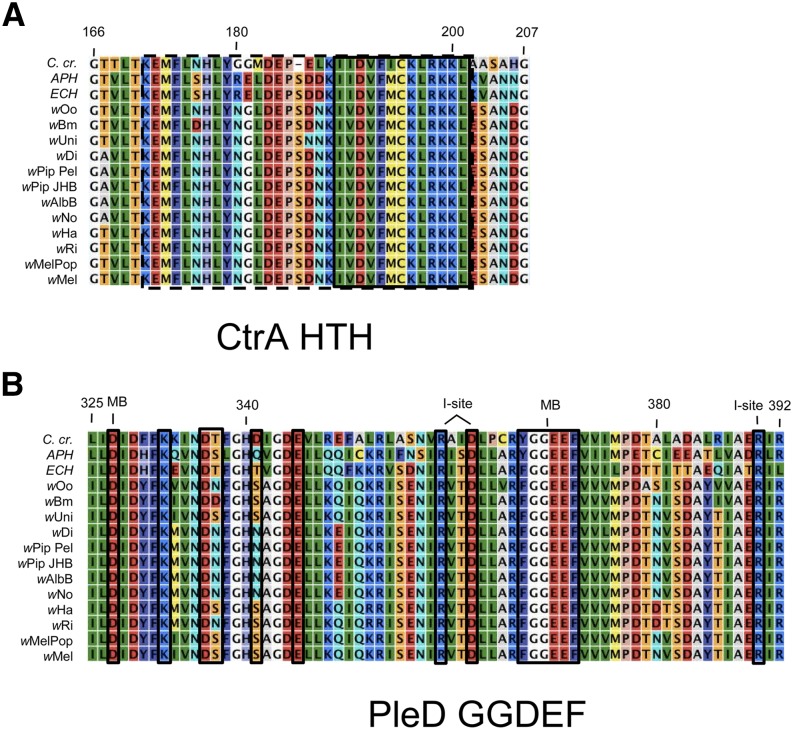
Analysis of CtrA and PleD output domain alignments. (A) CtrA HTH domain. (B) PleD GGDEF domain. Identical/similar residues are similarly colored. Amino acid numbers shown are for the sequence from *C. cresentus*. The HTH-DNA-binding motif in (A) is boxed with a dashed line, and the DNA-sequence-recognition α3 helix is boxed with a solid line. In the PleD GGDEF alignment in (B), the active site residues are boxed with a solid line and the positions of I-site and metal-binding (MB) residues are marked.

The response regulator CtrA was strikingly conserved in its domain structure between *C. crescentus*, *A. phagocytophilum*, *E. chaffeensis*, and *Wolbachia*. In all cases, CtrA was predicted to carry an N-terminal REC domain with a conserved Asp residue (Figure 3A, Figure S1, Table S5). The C-terminal helix-turn-helix (HTH) domain was also confirmed, and all *Wolbachia* strains carried the conserved α3-helical residues required for DNA binding (Figure 3A, Figure 5A) (Martinez-Hackert and Stock 1997; Quon *et al.* 1996; Lang and Beatty 2000; Bird and Mackrell 2011). This conservation suggests that the phospho-acceptor and DNA-binding properties of *Wolbachia* CtrA are analogous to CtrA in other α-proteobacteria. Analysis of seven *Wolbachia* genomes also identified 34 to 55 ORFs with upstream consensus CtrA binding sites, further supporting a role for *Wolbachia* CtrA *in vivo* (Table S7).

Predicted structural domains were also examined in PleC and PleD. *A. phagocytophilum* and *E. chaffeensis* PleC domain structure was similar to *C. crescentus* PleC, with predicted N-terminal transmembrane domains, an internal DHp domain, and a C-terminal CA domain, all carrying key functional residues, although no PAS or PAS-like domains were detected (Figure 3B, Figure 4C, Figure S1, Table S5, Table S6). The *Wolbachia* PleCs were similarly organized in nearly all strains, carrying a pair of transmembrane domains, an internal DHp domain, a C-terminal CA domain, and all key residues. PleC of *w*Pip JHB was distinctive in the loss of a transmembrane domain, and *w*Oo was, as noted, predicted to lack PleC altogether (Figure 3B). This suggests that most *Wolbachia* PleC proteins function similarly to PleC in other Anaplasmataceae (Kumagai *et al.* 2006; Lai *et al.* 2009).

The predicted domain structure of the PleD RR also appears widely conserved. As detected in *C. crescentus*, *A. phagocytophilum*, and *E. chaffeensis*, nearly all *Wolbachia* PleD proteins were predicted to carry two N-terminal REC domains with conserved Asp residues (Figure 3B, Table S5). One exception was *w*AlbB PleD, which carried an Asp-to-Tyrosine substitution in the internal REC domain. The other exception was *w*Oo PleD, in which the REC domains were separated by a split ORF, and the dissociated REC carried an Asp to Asn substitution. The GGDEF domain at the PleD C-terminus was also shared between *Wolbachia* and other Anaplasmataceae (Figure 3B). Twelve out of 14 key catalytic residues in the GGDEF were identical between all species and strains examined (Figure 5B) (Chan *et al.* 2004). Complete conservation was observed in all key residues of the GGDEF I-site, which is known to inhibit catalytic function in response to *c-di*-GMP binding (Christen *et al.* 2005; Christen *et al.* 2006). These results suggest that the majority of *Wolbachia* PleDs have similar functional and regulatory capacity as PleD of related bacteria.

### Analysis of cognate specificity residues in *Wolbachia* TCS proteins

The conservation of key functional domains in *Wolbachia* TCS proteins raises the question of whether they interact as exclusive functional pairs or are capable of cross-talk. Prior work comparing HK/RR pairs from 200 bacterial genomes has indicated a subset of residues that specify interaction within a cognate pair (Skerker *et al.* 2008; Capra *et al.* 2010). Nine residues in the HK DHp domain form a spatially constrained interface with seven residues in the REC domain of the cognate RR. Pairs of residues within this interface have been shown to co-vary between species. *In vitro* studies also show that mutating two to three residues in the HK DHp domain or three to four residues in the RR REC domain changes the specificity of HK/RR interaction (Skerker *et al.* 2008; Bell *et al.* 2010; Capra *et al.* 2010, 2012a). To assess the likelihood of exclusive CckA/CtrA and PleC/PleD interactions in *Wolbachia*, we examined the cognate specificity residues in these proteins through amino acid alignments with other Anaplasmataceae homologs informed with data from co-crystalized HK/RR pairs of major model systems (Casino *et al.* 2009; Capra *et al.* 2012a; Capra *et al.* 2010).

**Figure 6:**
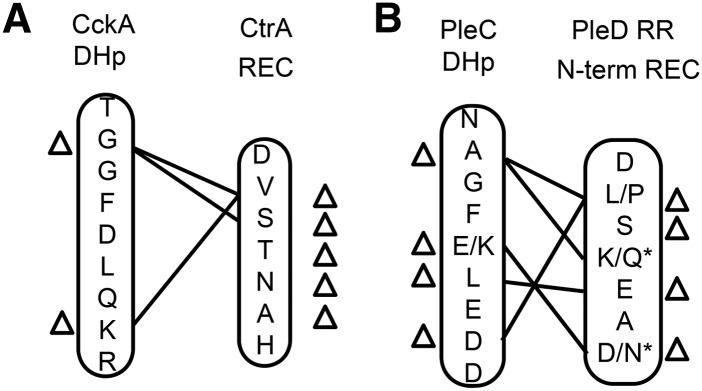
Comparison of co-evolving residues in cognate pairs from *Wolbachia*. Amino acid residues that specify cognate pairing for the (A) CckA/CtrA pair and the (B) PleC/PleD pair are listed. Change in *Wolbachia* sequences from *E. chaffeensis* identities are indicated by a neighboring triangle (Δ). *The majority of residues at that position are unchanged. Lines connecting HK and RR positions in the alignment indicate potential covariation for *Wolbachia* pairs corresponding with an “adjusted mutual information score” of higher than 3.5 using high-value pairing of canonical histidine kinases and response regulators (Capra *et al.* 2012a).

Analysis of CckA DHp cognate specificity residues revealed that seven out of nine key amino acids were identical between other Anaplasmataceae and *Wolbachia* (Figure 4A). Both of the nonhomologous amino acids in *Wolbachia* CckA were at positions known to co-vary in other species (Figure 6A) (Bell *et al.* 2010; Capra *et al.* 2010, 2012b). Furthermore, the amino acid identities of these key residues were identical in all *Wolbachia* strains (Figure 4A). By contrast, the cognate specificity residues of the CtrA REC domain displayed little homology between Anaplasmataceae and *Wolbachia*, with only two out of seven amino acid identities shared between the genera (Figure 4B). The majority of these nonconserved residues in *Wolbachia* CtrA were not explainable by covariation (Figure 6A). However, the identity of cognate specificity residues in CtrA was shared between all *Wolbachia* strains (Figure 4B). This indicates that, overall, CckA and CtrA residues that specify cognate pairing are highly conserved within *Wolbachia*. However, it is unclear whether they have retained an exclusive pairing affinity (Cheng *et al.* 2006; Kumagai *et al.* 2006).

We also investigated potential for specificity of *Wolbachia* PleC/PleD interaction. Compared against *E. chaffeensis* PleC, most *Wolbachia* PleC proteins were homologous at six out of nine cognate specificity residues in the DHp domain (Figure 4C). Supergroup B *Wolbachia* strains were distinct, showing homology at five out of nine residues (Table 1, Figure 4C). These divergent *Wolbachia* PleC residues corresponded to sites of predicted covariation (Figure 6B) (Capra *et al.* 2012a). By contrast, the PleD N-terminal REC domain was less conserved, with only two to four out of seven cognate specificity residues shared between *E. chaffeensis* and *Wolbachia* (Figure 4D). The nonhomologous residues varied along phylogenetic lines, with *w*Oo PleD of supergroup C showing the greatest divergence. Interestingly, the four positions with strongest potential for covariation did coincide with *Wolbachia* PleD polymorphisms (Figure 6B). These data indicate that PleC/PleD cognate specificity residues are less conserved between *Wolbachia* than those seen for CckA/CtrA. However, as divergence of *Wolbachia* PleC and PleD sequences could largely be explained by covariation, it remains possible that PleC/PleD function as a cognate pair.

## Discussion

**Figure 7:**
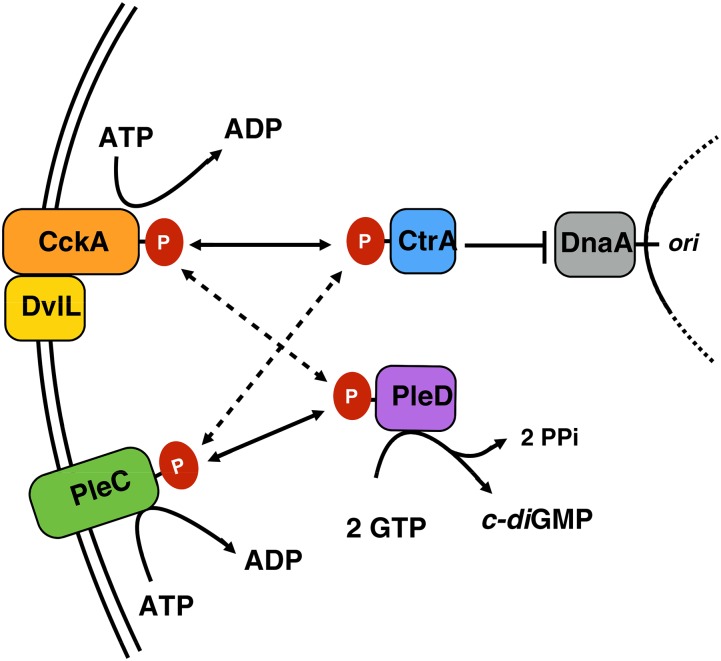
Model for interaction of TCS signaling proteins in *Wolbachia*. Interaction of HKs with RRs for most strains investigated in this study. Arrows indicate the predominant direction of phosphotransfer or substrate flow. CtrA output is represented as the inhibition of chromosome replication only.

This study has revealed that the core TCS factors CckA, CtrA, PleC, and PleD and several of their interacting proteins were conserved between *C. crescentus*, *A. phagocytophilum*, *E. chaffeensis*, and 12 sequenced *Wolbachia* strains. The genome-wide positioning of TCS genes was not well-conserved between *Wolbachia* or in relation to other Anaplasmataceae, in keeping with the extensive genomic rearrangements noted in other studies (Klasson *et al.* 2008, 2009; Wu *et al.* 2004). The immediate context of the core TCS loci was appreciably conserved, especially within host/supergroup divisions. Much of the domain structure and key functional residues of the predicted TCS proteins were conserved between *Wolbachia* strains and the other Anaplasmataceae, although cognate specificity residues between CckA/CtrA and PleC/PleD showed considerable divergence. This suggests that while these core TCS relays are generally retained in *Wolbachia*, there are important regulatory and functional differences in usage of *Wolbachia* TCS proteins relative to other characterized systems (Figure 7, Figure S1).

Extensive prior analysis of TCS genes has indicated that functional TCS pairs often occupy single operons (Laub and Goulian 2007), as is seen for 46 out of 106 TCS genes in *C. crescentu*s (Nierman *et al.* 2001; Skerker *et al.* 2005). This was not the case for *A. phagocytophilum*, *E. chaffeensis*, or *Wolbachia*. The condensation of genes flanking TCS ORFs in *Wolbachia* suggests distinctive regulatory streamlining. For example, *Wolbachia* TCS genes appear to share upstream regions with metabolic genes, such as *cckA* with *hemF*, and the *pleC* operon with the *argD* operon. Perhaps *Wolbachia* TCS gene expression benefits from consistent metabolic coupling in specific invertebrate host backgrounds, whereas the context of *Anaplasma* and *Ehrlichia* TCS genes provides more flexibility to adapt to changing host environments of tick, deer, and mammalian immune cells (Bakken and Dumler 2008; Jongejan and Uilenberg 2004).

The well-conserved domain structures of predicted *Wolbachia* TCS proteins highlight the functional importance of those domains. Given the nearly complete conservation between predicted CckA proteins, the *w*Alb CckA protein lacking a C-terminal REC domain stands out as a notable exception (Figure 3A). Prior studies have suggested that C-terminal REC domains of hybrid HKs serve as an “insulator” that prevents nondiscriminate phosphorylation of multiple RRs (Capra *et al.* 2012a,b). Thus, loss of the C-terminal REC is expected to lead to increased promiscuity and/or cross-talk, particularly in complex bacterial systems that carry dozens of TCS pairs (Laub and Goulian 2007). Perhaps the extremely low number of TCS proteins in *Wolbachia* endosymbionts reduces the requirement for an analogous insulatory function in the CckA hybrid HK.

The consistent detection of PAS-like domains in the predicted *Wolbachia* CckAs was also very striking. This groups *Wolbachia* CckA with a wide range of bacterial and eukaryotic PAS domain proteins, from redox-potential receptors in *E. coli* to human cardiac myocytes (Taylor and Zhulin 1999; Gu *et al.* 2000). Alignment of *Wolbachia* CckA to solved crystal structures further suggested that these PAS-like domains consistently associate with heme and may interact with FAD or FMN ligands as well. This invokes a conserved “sensor” capacity for CckA that could influence the potential for CckA-based regulation of the *Wolbachia* cell cycle.

The strong conservation of DvlL sequences between *Wolbachia* strains suggests an important functional role for this protein. *Wolbachia* DvlL was found to form three PAS-like folds, as also reported in *C. crescentus*, *A. tumefaciens*, and other species (Childers *et al.* 2014). Notably, DvlL of *Wolbachia* and the other Anaplasmataceae consistently lacked a C-terminal catalytic domain. Elegant experiments demonstrated that DivL catalytic activity is not required for regulation of the CckA-ChpT-CtrA pathway in *Caulobacter* (Reisinger *et al.* 2007; Iniesta *et al.* 2010). Thus, it is formally possible that DvlL affects CckA signaling function in the streamlined *Wolbachia* system as well (Figure 7). The close genetic association of *dvlL* with the *ctrA* locus in all *Wolbachia* genomes also suggests a conserved relationship that bears closer scrutiny. However, it cannot be ruled out that DvlL may have been repurposed for one or more other essential functions in *Wolbachia*.

Of all *Wolbachia* TCS proteins examined, CtrA showed the strictest conservation. As seen in *Caulobacter* and *E. chaffeensis*, dozens of *Wolbachia* genes also appear to be regulated by CtrA, including genes of diverse functional classes as well as *ctrA* itself (Table S7) (Laub *et al.* 2002; Cheng *et al.* 2011; Brilli *et al.* 2010). Conservation of *dnaA* in all *Wolbachia* strains analyzed also supports an important role for CtrA in regulating genome replication. A recent study analyzing eight strains of *Wolbachia* identified three DnaA binding sites and up to five CtrA consensus binding sites per *ori* (Ioannidis *et al.* 2007). These findings highlight CtrA as a “master regulator” of both gene expression and chromosome replication within the *Wolbachia* genus.

TCS domain comparisons highlight distinctions between the PleC sensing capacity in *Caulobacter* compared with the Anaplasmataceae. Although PleC is generally conserved between these species, no sensory PAS domains were detected in *A. phagocytophilum*, *E. chaffeensis*, or *Wolbachia* PleC (Cheng *et al.* 2006). It is possible that *Wolbachia* PleC functions in an unregulated manner. Because PleC contains residues essential for both kinase and phosphatase activity, its function may also be heavily influenced by ATP availability. It is also possible that Anaplasmataceae PleC senses periplasmic cues through non-PAS structural features or is regulated by factors associated with the plasma membrane, as has been shown in *Caulobacter* (Paul *et al.* 2008; Smith *et al.* 2012).

Insights into *Wolbachia* PleD function are also suggested by variation in the PleD REC domains of two *Wolbachia* strains. Previous work suggests that the PleD N-terminal REC is mainly responsible for regulating PleD GGDEF activity (Aldridge *et al.* 2003; Lai *et al.* 2009). If this paradigm extends to *Wolbachia*, loss of an Asp residue from the internal REC domain of *w*AlbB PleD may have little functional impact. In *w*Oo PleD, however, the original N-terminal REC lacks this key Asp residue and is further predicted to be physically separate from the fully conserved GGDEF domain. In this case, the remaining REC domain may regulate the GGDEF, analogous to the WspR protein in *P. aeruginosa* (De *et al.* 2008, 2009). Alternatively, the I-site that downregulates GGDEF activity in response to *c-di*-GMP binding may have a primary regulatory role (Chan *et al.* 2004; De *et al.* 2008; Lai *et al.* 2009). Conservation of I-site functional residues in all *Wolbachia* PleDs, including *w*Oo, is consistent with this possibility. Because the complexity of second messenger signaling by *c-di*-GMP has been unaddressed in *Wolbachia* and many other symbiotic bacteria, this remains a poorly understood area of host–microbe interaction studies.

Analysis of the key residues that specify pairing between TCS proteins has also shown differences between *Wolbachia* and other systems including *A. phagocytophilum* and *E. chaffeensis* (Capra and Laub 2012; Capra *et al.* 2010; Cheng *et al.* 2006; Kumagai *et al.* 2006; Lai *et al.* 2009). Although the overall amino acid identity of *Wolbachia* CckA largely matched those of *Ehrlichi*a, *Wolbachia* CtrA, PleC, and PleD cognate specificity residues varied extensively, consistent with the potential for loss of HK/RR interaction specificity of other systems (Capra *et al.* 2010; Bell *et al.* 2010). Surprisingly, the identity of nearly every cognate specificity residue was conserved between *Wolbachia* strains. Perhaps CckA/CtrA and PleC/PleD have co-evolved in a manner that preserved spatially constrained, specific interactions between these TCS pairs. An alternative explanation is that cross-talk is common and necessary in the streamlined *Wolbachia* system (Figure 7). Future experiments are needed to determine the absolute requirements for TCS regulation of *Wolbachia* in the context of the host environment. Together, work on this important endosymbiont and its divisional regulation will help to inform the mechanisms underlying *Wolbachia* titer regulation and interactions between *Wolbachia* and host.
